# Surface-Engineered HA-PEG-ICG/PLGA Nanoprobes with Vessels Targeting for Lymphatic System Visualization

**DOI:** 10.1021/acsabm.5c00769

**Published:** 2025-08-27

**Authors:** Hao-Han Chiang, Yu-Teng Chang, Wei-Ren Huang, Min-Xuan Cai, Chin-Hsing Feng, Jia-Ning Syu, Chih-Sheng Lai, Yi-Hsin Chien

**Affiliations:** † Department of Materials Science and Engineering, 34902 Feng Chia University, Taichung 40724, Taiwan; ‡ Division of Plastic and Reconstructive Surgery, Department of Surgery, 40293 Taichung Veterans General Hospital, Taichung 40705, Taiwan; § Department of Post-Baccalaureate Medicine, College of Medicine, 124702 National Chung Hsing University, Taichung 40227, Taiwan; ∥ Division of Nephrology, Department of Internal Medicine, Taichung Veterans General Hospital, Taichung 40705, Taiwan

**Keywords:** lymphatic imaging, ICG/PLGA nanocarrier, indocyanine green, hyaluronic acid, targeted fluorescence probe, lymphedema diagnostics

## Abstract

Targeted imaging of the lymphatic system is essential for the early diagnosis and management of lymphatic disorders, such as lymphedema. In this study, we developed a lymphatic-targeted fluorescent nanoprobe by encapsulating indocyanine green (ICG) within poly­(lactic-*co*-glycolic acid) (PLGA) nanoparticles, further surface-modified with hyaluronic acid-polyethylene glycol (HA-PEG) to enhance specificity (HA-PEG-ICG/PLGA NPs). The nanoparticles were synthesized *via* a microemulsion technique followed by surface cross-linking, and thoroughly characterized by ultraviolet–visible (UV–vis) spectroscopy, fluorescence emission analysis, Fourier transform infrared (FTIR) spectroscopy, and ζ-potential measurements, confirming their physicochemical stability and functionalization. *In vitro* cytotoxicity assays indicated excellent biocompatibility with both human keratinocytes (HaCaT) and mouse lymphatic endothelial cells (SVEC4–10). Confocal microscopy and quantitative fluorescence analyses revealed significantly enhanced uptake of HA-PEG-ICG/PLGA NPs in SVEC4–10 cells, which was attributed to HA-mediated binding to LYVE-1 receptors. *In vivo* imaging in C57BL/6JCrlBltw mice further demonstrated prolonged retention and selective fluorescence accumulation in lymphatic vessels following intraperitoneal administration, surpassing those of free ICG and ICG/PLGA controls. Collectively, these results confirm the potential of HA-PEG-ICG/PLGA NPs as a safe and effective nanoplatform for real-time lymphatic imaging. This targeted system holds promises for early lymphedema diagnosis, intraoperative lymphatic mapping, and future integration with theragnostic strategies for lymphatic-associated diseases.

## Graphical abstract

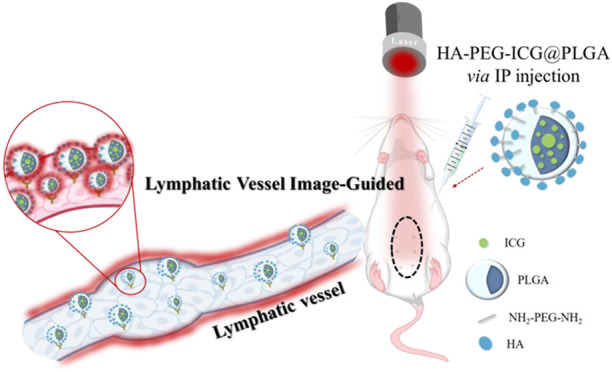

## 1 Introduction

Limb lymphedema is a chronic disease and affects over 200 million individuals globally at various stages, significantly diminishing their quality of life.^1^ Any type of problem that blocks the drainage of lymph fluid can cause lymphedema. This condition is predominantly associated with cancer treatments, particularly those employed in the management of breast cancer. Research indicates that 21% of breast cancer survivors experience complications related to lymphedema following their recovery.^2^ Moreover, patients who undergo axillary lymph node dissection are at a 4-fold increased risk of developing lymphedema.^3^ Additionally, postoperative radiation therapy has been shown to elevate the risk of lymphedema by a factor of 10.^4^ It is a noteworthy concern that lymphedema resulting from cancer treatment may not become apparent until several months or even years after the cessation of therapy. The signals and symptoms of lymphedema can range from mild to severe, including swelling of part or all of the arm or leg, feeling of heaviness or tightness, affecting the ability to move, recurring infections, and hardening and thickening of the skin (fibrosis). These external symptoms may not directly develop in a short period of time, leading to delays in seeking medical treatment. Therefore, the International Society of Lymphology (ISL) provides a classification standard of lymphedema based on its severity into four stages: Stage 0 (Incubation period), Stage I (Mild), Stage II (Moderate), and Stage III (Severe).^2^ Among them, conservative therapy is effective in the status of lymphedema patients at stages 0 and I to improve lymphatic drainage and reduce fluid accumulation. For moderate or further severe cases, where fibrosis and pain occur, additional approaches such as low-dosage laser therapy and surgical interventions are needed, including liposuction, lymphatic vessel transplantation, lymphatic-venous anastomosis (LVA), and vascularized lymph node transfer (VLNT), which is aimed at restoring or bypassing damaged lymphatic pathways to improve fluid drainage and limb function.^5–7^

In clinical trials, LVA has emerged as a promising therapeutic approach for stage III lymphedema. This method employs a near-infrared (NIR) fluorescence agent to improve the visualization of lymphatic vessels. Among clinically available agents, indocyanine green (ICG) remains a widely adopted NIR probe due to its FDA approval and established safety profile. ICG has been extensively utilized for intraoperative fluorescence imaging, including LVA, providing surgeons with real-time visualization to improve surgical precision and outcomes, this process is referred to as ICG lymphography.^8^ Notably, it demonstrates high sensitivity, enabling the detection of early or latent-stage lymphedema.^9^ Intraoperatively, this technique provides real-time imaging of the lymphatic system, which can aid surgeons in assessing lymphatic circulation prior to and during lymphatic surgical procedures, such as LVA, thus informing surgical decision-making.^10^ However, in patients with moderate to severe lymphedema, there may be considerable leakage of lymph fluid, which can lead to the concomitant leakage of ICG. This phenomenon may result in imaging that presents as stardust or diffuse patterns, thereby complicating the physician’s ability to accurately identify the locations of the lymphatic vessels.^11^ Consequently, surgeons may need to rely on anatomical knowledge and intraoperative experience. Given the considerable interindividual variability in lymphatic anatomy, this reliance introduces uncertainty and highlights the need for improved contrast agents or complementary imaging techniques.

Despite its clinical approval and widespread use, ICG is limited by several intrinsic drawbacks, including rapid photobleaching, a short circulatory half-life (∼2–4 min), and a lack of inherent targeting capability, all of which hinder its broader application in precision diagnostics.^12^ Recent advancements in nanotechnology, particularly in the development of surface-engineered nanomaterials and NIR emissive nanoprobes, have addressed many of the limitations associated with conventional imaging agents.^12^ Emerging NIR-II probes, such as donor–acceptor (D–A) conjugated polymers,^13^ cyanine dye derivatives,^14^ and polymethine-based fluorophores,^15^ have demonstrated tunable emission profiles, superior photostability, and high biocompatibility, making them ideal for deep-tissue imaging, including applications in lymphatic and microvascular visualization.^16^ In particular, surface-modified nanoparticles (NPs) functionalized with targeting ligands provide a versatile and efficient platform for the delivery of diagnostic and therapeutic agents to the lymphatic system.^17^ Lymphatic-specific receptors such as lymphatic vessel endothelial hyaluronic acid receptor 1 (LYVE-1),^18^ scavenger receptor class B member 1 (SR-B1),^19^ and vascular endothelial growth factor receptor 3 (VEGFR-3)^20^ have been identified as viable molecular targets for lymphatic imaging. Consequently, nanoparticles conjugated with synthetic ligands that exhibit a high affinity for these receptors can facilitate improved localization to lymph nodes and enhanced uptake by lymphatic endothelial cells. Moreover, small-molecule ligands such as hyaluronic acid (HA) and DEC-205 have been employed to functionalize nanoparticle surfaces to selectively target immune cells, including macrophages (*via* CD44) and dendritic cells (*via* CD205), respectively.^21,22^

In order to overcome the limitations associated with ICG and to augment its applicability in lymphatic imaging of lymphedema. In this study, we developed a multifunctional fluorescent nanoprobe, HA-PEG-ICG/PLGA, by encapsulating ICG within poly­(lactic-*co*-glycolic acid) (PLGA) nanoparticles and functionalizing the surface with polyethylene glycol (PEG) and hyaluronic acid (HA). The incorporation of HA facilitates targeted delivery through CD44 receptor-mediated endocytosis, whereas PEG enhances colloidal stability and prolongs systemic circulation time. The efficacy of HA-PEG-ICG/PLGA was evaluated in relation to its targeting capabilities on human keratinocyte cells (HaCaT) and mouse lymph node endothelial cells (SVEC4–10). Furthermore, an *in vivo* assessment of biosafety, histological examination *via* H&E staining, and lymphatic vessel targeting were conducted through interactions with female C57BL/6 mice. This structural approach significantly improves the optical performance, biocompatibility, and lymphatic specificity of ICG, thereby rendering it more suitable for applications in lymphatic imaging and image-guided surgical procedures (as seen in Scheme 1).

**Scheme 1:**
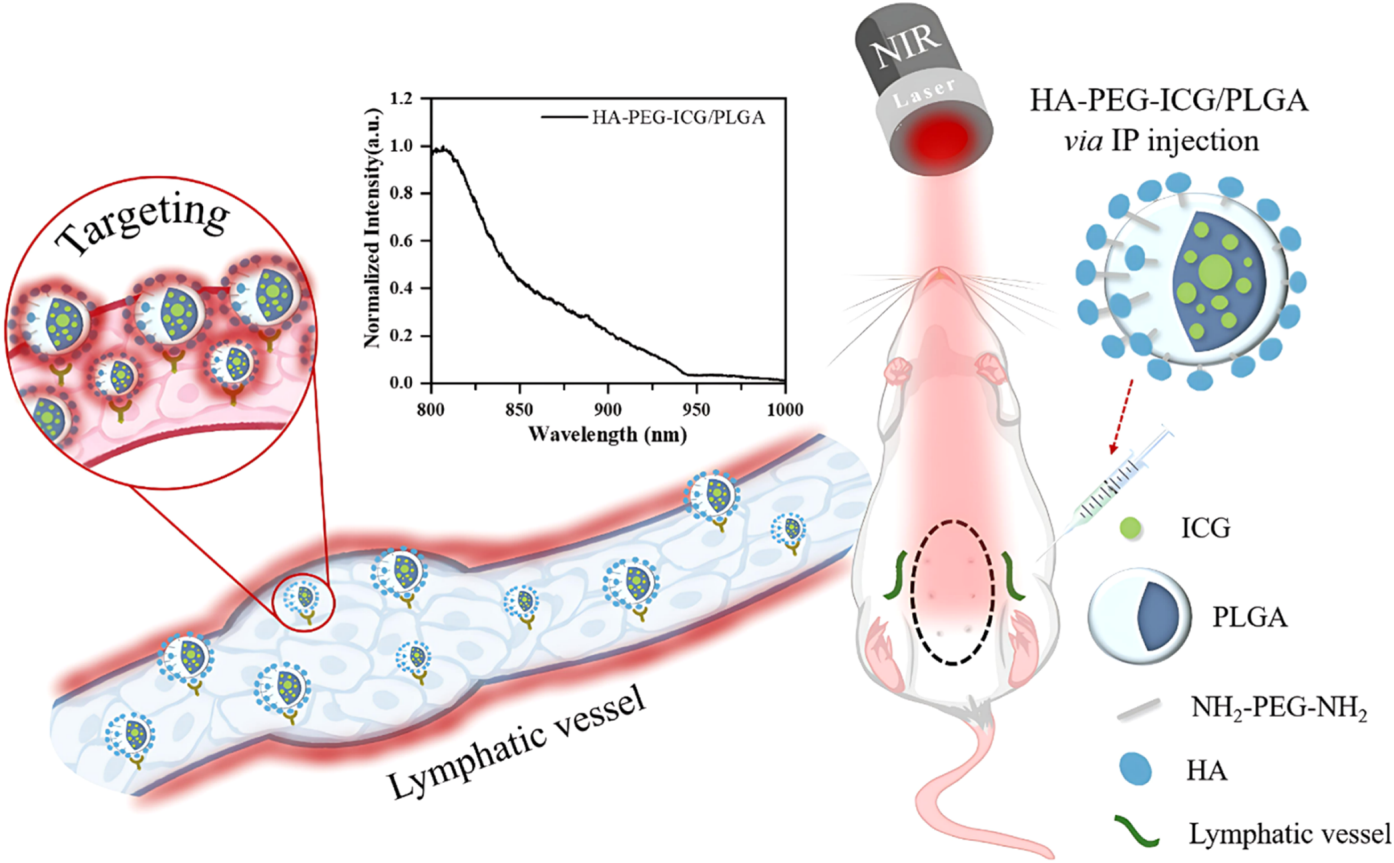
Biocompatible ICG-Loaded PLGA Nanoparticles Functionalized with HA-PEG for Specificity Enhanced Lymphatic Targeting through Fluorescence Spectrum and Confocal Image in *In Vitro* and *In Vivo* Study.

## 2 Materials and Methods

### 2.1 Materials

Deionized (DI) water with a resistivity of 18.23 MΩ cm^–1^ was produced by RODA, laboratory-grade NRD (Te Chen Instruments Co., Ltd., Taichung, Taiwan). Indocyanine green (ICG, *M*_w_ = 774.96) was purchased from Taiyo Pharma Tech Co., Ltd. (Tokyo, Japan). Methanol (CH_3_OH) was purchased from Duksan Pure Chemicals (Ansan, Korea). Poly­(vinyl alcohol) (PVA, *M*_w_ = 61000), *N*-hydroxy succinimide (NHS, *M*_w_ = 115.09), and Hyaluronic acid (HA, *M*_w_ = 1.5–1.8 × 10^6^) were purchased from Sigma-Aldrich (St. Louis, MO, America). Dichloromethane (DCM), 1-(3-dimethylaminopropyl)-3-ethylcarbodiimide (EDC, *M*_w_ = 155.245), Poly­(ethylene glycol) bis­(amine) (NH_2_–PEG-NH_2_, *M*_w_ = 3400) was purchased from Thermo Fisher Scientific (Waltham, MA, America). Poly­(lactic-*co*-glycolic acid) (PLGA, *M*_w_ = 7000–17,000) was purchased from Polysciences, Inc. (Norristown, PA, America).

### 2.2 Synthesis of PLGA Nanoparticles Encapsulating ICG (ICG/PLGA NPs)

The poly­(lactic-co-glycolic acid) nanoparticles encapsulating indocyanine green (ICG/PLGA NPs) were prepared using the microemulsion method. The details of the synthesis process are shown in **Figure S1**. The microemulsion method involves the formation of a transparent or translucent thermodynamically stable system of water, oil, surfactant, and cosurfactant in appropriate proportions. This system has an extremely low interfacial tension and does not show phase separation even after long-term storage. Depending on the order of adding the oil phase and water phase, it can form either a W/O (water-in-oil) or an O/W (oil-in-water) type microemulsion. The following is the experimental setup method.

First, the water phase (W phase) solution was prepared by adding 2 g of PVA powder to 100 mL of DI water to create a 2% PVA solution. The mixture was heated to 78 °C, stirred until the powder was completely dissolved, and then allowed to cool naturally to room temperature. Next, the oil phase (O phase) solution was prepared by dissolving 30 mg of PLGA powder in 1.4 mL of DCM to form oil phase solution A. Then, 0.3 mL (1.5 mM) of ICG solution was dissolved in 0.3 mL of methanol to prepare oil phase solution B. Finally, solutions A and B were mixed uniformly.

The uniformly mixed O phase solution was slowly added to the W phase solution, and the mixture was sonicated in a sonicator (DELTA, D9NX-DC200H) for 30 min. Afterward, the solution appeared as a uniform, milky green gel. The solution was then stirred with a magnetic stirrer at 400 rpm for 4 h in the dark to allow DCM to evaporate and form ICG/PLGA NPs. The solution was centrifuged three times at 6200 Xg for 15 min each using a high-speed centrifuge (Thermo Fisher Pico21) to remove unencapsulated ICG molecules and other unreacted compounds. Finally, DI water was added to collect the ICG/PLGA NPs.

### 2.3 Synthesis of NH_2_–PEG-ICG/PLGA NPs

The amine-functionalized poly­(ethylene glycol) (NH_2_–PEG-NH_2_) molecules are modified on the surface of ICG/PLGA NPs to facilitate subsequent conjugation with hyaluronic acid (HA), which has a high specificity for binding to lymphatic endothelial cells. The details of the synthesis process are shown in Figure S2. A chemical cross-linking reaction is conducted using EDC and NHS to catalyze the formation of an amide bond (−CONH_2_) between the carboxyl group (−COOH) on the surface of PLGA NPs and the amine group (−NH_2_) in NH_2_–PEG-NH_2_. This process results in the surface modification of ICG/PLGA NPs with NH_2_–PEG-NH_2_ molecules.^23^ First, EDC powder and NHS powder (EDC/NHS molar ratio of 1:2) were dissolved in DI water. Then, according to the ratio (ICG/PLGA/EDC/NHS molar ratio = 1:10:20), the corresponding amount of ICG/PLGA NPs solution was taken and mixed for the reaction. The EDC solution was mixed with the ICG/PLGA NPs solution and stirred with a magnetic stirrer at 800 rpm for 10 min in the dark. Next, the NHS solution was added, and the mixture was stirred at 800 rpm for 20 min in the dark. After that, the NH_2_–PEG-NH_2_ solution (3.4 mg of powder dissolved in 0.1 mL of DI water) was added to the mixture, and stirring continued at 800 rpm for 4 h in the dark. This allowed the amine groups in the NH_2_–PEG-NH_2_ molecules to cross-link with the activated carboxyl groups on the surface of the PLGA nanoparticles, forming stable covalent bonds.

Finally, the mixture was centrifuged three times at 6200 Xg for 15 min each using a high-speed centrifuge to remove any unmodified PEG molecules. Afterward, DI water was added to collect the precipitate, resulting in NH_2_–PEG-ICG/PLGA NPs.

### 2.4 Synthesis of HA-PEG-ICG/PLGA NPs

A chemical cross-linking reaction is carried out using EDC and NHS to catalyze the formation of an amide bond between the carboxyl groups in HA and the exposed amine groups on the surface of NH_2_–PEG-ICG/PLGA NPs. This modification attaches HA, which specifically targets lymphatic endothelial cells, to the surface of NH_2_–PEG-ICG/PLGA NPs, enabling the labeled imaging of lymphatic vessels. The details of the synthesis process are shown in Figure S3. First, EDC powder and NHS powder (EDC/NHS molar ratio = 1:2) were dissolved in DI water. The EDC solution was mixed with the HA aqueous solution (0.5 mg/mL) and stirred with a magnetic stirrer at 800 rpm for 10 min in the dark. Then, the NHS solution was added, and the mixture was stirred at 800 rpm for 20 min in the dark. Finally, the resulting solution was mixed with the previously prepared NH_2_–PEG-ICG/PLGA NPs solution and stirred at 800 rpm for 4 h in the dark.

After the reaction was complete, the mixture was centrifuged 3 times at 6200 Xg for 15 min each using a high-speed centrifuge to remove unmodified HA molecules and other unreacted compounds. Then, DI water was added to collect the precipitate, resulting in HA-PEG-ICG/PLGA NPs.

### 2.5 Morphological Characterization of SNPs

Transmission electron microscopy (TEM) was used for the morphological study of the prepared particles. Ten μL of the sample was deposited onto a 200-mesh carbon-coated copper grid and was air-dried at room temperature. The samples were observed by TEM (JEOL, JEM-1400-FLASH) at an operating voltage of 120 kV. A Nanoparticle size and ζ-potential analyzer (Anton Paar, Litesizer-500) was used to analyze the hydrodynamic size distribution of fabricated nanoparticles. For these measurements, the pellets of nanoparticles were resuspended in 3 mL of Deionized water. DLS measurements were performed at 25 °C. The approximate net charge on the nanoparticle surface was calculated by Zeta (ζ) potential using the nanoparticle size and ζ-potential analyzer.

### 2.6 Fluorescence Measurements

A photoluminescence spectrometer (Andor-Oxford Instruments, Kymera 328i) was used to measure the fluorescence emission of the samples. Fluorescence spectra were taken in the wavelength range of 800–1000 nm with an excitation wavelength of 785 nm and 100 μm excitation and emission slit widths.

### 2.7 *In Vitro* Cell Culture Study

#### 2.7.1 Cell Culture

Human keratinocytes (HaCaT) and mouse lymph node endothelial cells (SVEC4–10), purchased from Cellverse Co., Ltd. (Shanghai, China) and the Food Industry Research and Development Institute (Hsinchu, Taiwan), respectively. Both cell lines were maintained in Dulbecco’s Modified Eagle Medium (DMEM, Thermo Fisher Scientific, Waltham, MA) supplemented with 10% fetal bovine serum (FBS, HyClone, Cytiva, Logan, UT), 1% penicillin/streptomycin/amphotericin B (PSA, VivaCell, Shanghai, China), and 4 mM l-glutamine (VivaCell). The cells were incubated at 37 °C in a humidified atmosphere with 5% CO_2_.

#### 2.7.2 Cell Viability

The cytotoxicity of HA-PEG-ICG/PLGA nanoparticles (NPs) on HaCaT and SVEC4–10 cells was evaluated by using the Cell Counting Kit-8 (CCK-8) assay (Addkine, Atlanta, GA). HaCaT and SVEC4–10 cells were seeded at a density of 1 × 10^4^ cells per well into 24-well plates. After cell adhesion, HA-PEG-ICG/PLGA NPs dissolved in DMEM were added to the wells at ICG concentrations of 5.3, 10.5, 21, 42.1, and 84.2 μM. The cells were then incubated for 24 h. After incubation, the supernatant was removed, and the cells were washed three times with PBS. Fresh DMEM containing 10% CCK-8 reagent was subsequently added to each well, followed by incubation for 1 h. Finally, 0.1 mL of the supernatant was collected, and the absorbance was measured at 450 nm by using a spectrophotometric reader (BMG SPECTROstar Nano, Ortenberg, Germany).

### 2.8 *In Vitro* Cellular Uptake and Bioimaging Study

HaCaT and SVEC4–10 cells were seeded at a density of 1 × 10^4^ cells per well in an 8-well chamber slide (ibidi, Gräfelfing, Germany). After cell adhesion, HA-PEG-ICG/PLGA NPs dissolved in DMEM were added to each well at ICG concentrations of 10.5, 21, 42.1, 63.2, and 84.2 μM. The chamber was incubated at 37 °C in a humidified atmosphere containing 5% CO_2_ for 24 h. Following incubation, the supernatant was removed, and the cells were washed three times with PBS. Fixation was performed in the dark for 20 min at room temperature using a fixation buffer (BioLegend, Cat# 420801). To permeabilize the cells, 0.1% Triton X-100 in PBS was applied for 3–5 min, followed by 2–3 washes with PBS. For staining, 200 μL of a 1× phalloidin conjugate working solution containing DAPI was added to each well. The staining solution was prepared by mixing 20 μL of DAPI Fluoromount-G (Abcam, Cambridge, U.K.), 1 μL of Phalloidin-iFluor 488 reagent (Abcam), and PBS to a final volume of 1 mL. Cells were incubated at room temperature for 60 min and gently washed 2–3 times with PBS to remove excess staining reagent. Mounting medium was then added, and the chamber was sealed. Fluorescence images were acquired by using a confocal laser scanning microscope (FLUOVIEW FV3000, Olympus, Tokyo, Japan).

### 2.9 *In Vivo* study

**Mice:** The care of animals adhered to the Laboratory Animal Welfare Act and the Guidelines for the Care and Utilization of Laboratory Animals, receiving approval from the Institutional Animal Care and Use Committee (IACUC) at Taichung Veterans General Hospital (TCVGH). Every animal treatment and surgical procedure followed the protocols outlined by the TCVGH Laboratory Animal Center (IACUC NO. La-1142145-V1). The experimental mice were kept in cages under conditions of 22 ± 2 °C temperature and 50 ± 70% humidity, following a light/dark cycle of 13 h/11 h.

#### 2.9.1 Biosafety (Hematoxylin and Eosin, H&E) Staining and Bioimage of Lymphatic Vessels

Female C57BL6/6JCrlBltw mice aged 4–6 weeks received either 400 μL of sterile PBS (control) and 400 μL of 6.45 μM (dissolved in sterile PBS) of ICG, ICG/PLGA NPs, and HA-PEG-ICG/PLGA NPs through Intraperitoneal (IP) Injections. After a 2-h systemic circulation period, experimental mice were sacrificed, and samples of normal organs (heart, liver, and kidney) were collected for H&E staining. The normal organ samples, including the heart, liver, and kidney, were embedded in paraffin and sliced into 5 μm thickness. The sections underwent deparaffinization, rehydration, PBS washing, and staining with hematoxylin solution (Merck) for 3 min. After rinsing in tap water, an eosin solution (Merck) was applied for 1 min. Subsequently, the sections were immersed in ethanol and xylene before being mounted for evaluation. The sections were examined under a BX51 microscope (Olympus), capturing images from three different fields for each group. Subsequently, lymphatic imaging has been addressed by the following process, the lymphatic vessels were directly removed and mounting media was added, and the chamber was sealed. Finally, fluorescence images were captured using a confocal laser scanning microscope (Olympus FLUOVIEW FV3000).

## 3 Results and Discussion

### 3.1 Characterization of ICG Encapsulated PLGA Nanoparticles (ICG/PLGA NPs)

As shown in Figure 1A, the ultraviolet–visible (UV–vis) absorption spectrophotometer has been applied to measure the ICG molecule, showing absorbance peaks at 715 and 780 nm, respectively. These two absorption peaks represent the different stacking molecule structure of ICG to the formation of monomer and dimers.^24,25^ Accordingly, the absorbance peaks of ICG/PLGA NPs demonstrate a slight red shift to 720 and 788 nm, attributed to the amount of stacking of ICG molecules into PLGA nanocarriers. The ICG molecule exhibits amphiphilic properties, consisting of polycyclic moieties linked by a long carbon chain. The rotation along the π-conjugated backbone of the cyanine dye has the potential to induce a red shift in both absorption and fluorescence peaks.^26^ Besides, there is no apparent absorption peak of PLGA NPs, indicating the successfully encapsulated ICG molecule into PLGA NPs through a microemulsion method. To determine the encapsulation efficiency (EE, %) of the ICG molecule in ICG/PLGA NPs, a concentration calibration curve for ICG was created by monitoring the intensity of the absorption peak at 780 nm across a range of ICG concentrations. The linear regression equation is *y* = 0.0594*x* + 0.1245, with a correlation coefficient of *R* ^2^ = 0.9885 (Figure S4). According to Beer’s Law, the encapsulation concentration of ICG in ICG/PLGA nanoparticles is positively correlated with absorbance intensity, as determined by a calibration curve. The encapsulation efficiency (EE, %) can be calculated by dividing the ICG encapsulation concentration by the total ICG concentration in the stock solution. The average encapsulation efficiency (EE, %) in this study is approximately 90%. Furthermore, we provide photoluminescence (PL) results to confirm the fluorescence optical properties of ICG molecules and ICG/PLGA NPs, as seen in Figure 1B. Upon irradiation with a 785 nm laser, the maximum emission peak of ICG molecules and ICG/PLGA NPs is observed at 806 nm. Additionally, certain stacking structures of ICG molecules exhibit a shoulder emission peak at 885 nm. The TEM images of PLGA NPs and ICG/PLGA NPs exhibit spherical morphology with particle sizes of 253.88 nm ± 59.91 nm and 290.58 nm ± 78.39 nm, respectively (Figure 1C,1D). In addition, the results of the dynamic light scattering (DLS) analysis for PLGA and ICG/PLGA NPs are presented in Figure S5. The average hydrodynamic diameters were found to be 260.9 and 389.2 nm, respectively.

**Figure 1:**
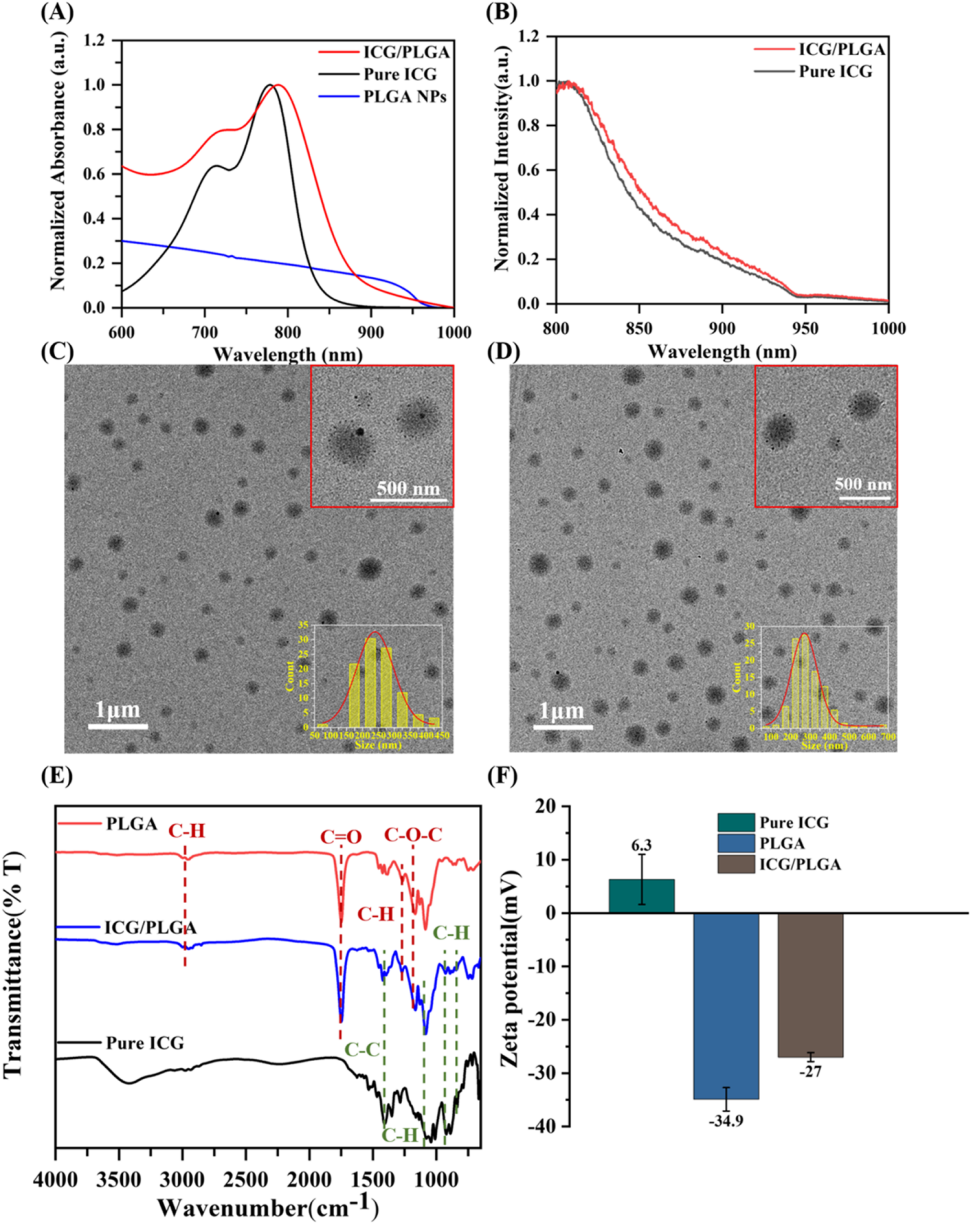
Optical characterization of Pure ICG, PLGA, and ICG/PLGA (dissolved in DI water) (A) normalized UV–vis spectrum; (B) normalized photoluminescence spectrum (under 785 nm laser excitation). The TEM image with size distribution insert of (C) PLGA; (D) ICG/PLGA. The surface behavior characterization of Pure ICG, PLGA, ICG/PLGA (E) FTIR spectra; (F) ζ-potential.

The purpose of the Fourier transform infrared (FTIR) was to provide evidence of surface functional groups on ICG/PLGA NPs, confirming the encapsulation of ICG within PLGA NPs, as illustrated in Figure 1E. In comparison to the pure ICG molecule, PLGA NPs, and the ICG/PLGA NPs, the FTIR spectrum of the ICG/PLGA NPs (blue line) and pure ICG (black line) exhibits similar characteristic bands at 836, 1085, and 1410 cm^–1^, which are assigned to C–H out-of-plane bending, C–H vinyl stretching, and aromatic C–C stretching, respectively.^27,28^ The characteristic bands of the ICG/PLGA NPs at approximately 1175, 1390 to 1440, 1760, and 3000 cm^–1^ correspond to the C–O–C stretching, C–H bending, C=O stretching, and C–H stretching of PLGA NPs, respectively.^29^ The ζ-potential analysis presented in Figure 1F provides evidence of the surface potential of pure ICG, PLGA, and ICG/PLGA, resulting in values of +6.3, −34.9, and −27 mV, respectively. Although ICG contains sulfonate groups contributing to a net negative charge, its overall surface charge can appear positive. This is due to the presence of other components, such as quaternary ammonium groups (NR_4_+) and the nature of surface-exposed moieties. The PLGA NPs exhibit a negative surface charge due to the presence of carboxyl groups (R-COOH) on their surface. Similarly, the ζ-potential of ICG/PLGA NPs demonstrates a slightly less negative value than that of PLGA NPs, attributed to the positive surface charge of pure ICG, indicating the successful encapsulation of ICG within the PLGA NPs.

### 3.2 Characterization of PEGylated ICG@PLGA NPs and HA Modified PEG-ICG@PLGA NPs

Hyaluronic acid (HA) represents a significant approach to enhancing the specific targeting of lymphatic endothelial cells. HA molecule is modified onto the ICG/PLGA NPs to improve targeting performance. First, amino-functionalized polyethylene glycol (NH_2_–PEG-NH_2_), a homobifunctional PEG derivative, can be utilized as a linker between the ICG/PLGA NPs and HA. Due to the presence of carboxyl groups on the outer surface of the PLGA NPs and HA molecule; the amino groups in the PEG molecule, forming the amide bond linkage through an EDC/NHS carbodiimide chemistry.^30,31^ As illustrated in Figure 2, the materials characterization of PEGylated ICG/PLGA NPs (NH_2_–PEG-ICG/PLGA NPs) and HA-PEG-ICG/PLGA NPs were conducted through the analysis of UV–vis spectra, PL spectra, FTIR, SPSA, and TEM images. As seen in Figure 2A, the absorption peak of HA-PEG-ICG/PLGA NPs, observed at 744 and 843 nm, exhibits a slight red shift compared to NH_2_–PEG-ICG/PLGA NPs (λ_abs_ = 825 nm). Furthermore, their emission peaks are recorded at 806 and 811 nm, respectively, which are consistent with the emission properties of ICG (Figure 2B). The FTIR analysis provides successful evidence of surface modification in NH_2_–PEG-ICG/PLGA NPs and HA-PEG-ICG/PLGA NPs, as seen in Figure 2C. The characteristic peaks in NH_2_–PEG-ICG/PLGA NPs correspond to N–H stretching, CH_2_ stretching, and C–O stretching at 3528, 2884, and 1103 cm^–1^, respectively, indicating successful surface modification by NH_2_–PEG-NH_2_.^32^ Furthermore, the characteristic peaks in HA-PEG-ICG/PLGA NPs exhibit an O–H stretching in the range of 3200 to 3500 cm^–1^, which also indicates successful surface modification by HA molecules. Additionally, the characteristic peaks in both NH_2_–PEG-ICG/PLGA NPs and HA-PEG-ICG/PLGA NPs at 1453, 1653, and 1762 cm^–1^ are assigned to C–N stretching, N–H bending, and C=O stretching, respectively, which are attributed to the presence of amide bonds. These findings suggest that NH_2_–PEG-NH_2_ serves as a bridge to conjugate ICG/PLGA NPs and HA molecules through the EDC/NHS carbodiimide chemical reaction. The ζ-potential profile serves as a critical tool for assessing the alteration in surface charge, thereby illustrating the behavior of surface modification. A surface charge analysis was conducted on NH_2_–PEG-NH_2_, NH_2_–PEG-ICG/PLGA NPs, HA, and HA-PEG-ICG/PLGA NPs, as depicted in Figure 2F. The ζ-potential of NH_2_–PEG-NH_2_ was recorded at −0.83 mV, while that of ICG/PLGA NPs was −27 mV. Following the modification of NH_2_–PEG-NH_2_ onto the surface of ICG/PLGA NPs to create NH_2_–PEG-ICG/PLGA NPs, an amide bond was formed between the hydroxyl group on the carboxyl group (R-COOH) of the PLGA NPs and the amine group on the PEG. This modification resulted in a shift in ζ-potential from −27 to −24.3 mV. The ζ-potential of HA was measured at −7.3 mV, while HA-PEG-ICG/PLGA NPs exhibited a ζ-potential of −27.4 mV. This alteration can be attributed to the negative charges associated with the hydroxyl groups present on the HA molecules. Following modification with HA, the ζ-potential of the nanoparticles transitioned from −24.3 to −27.4 mV. The particle sizes of NH_2_–PEG-ICG/PLGA NPs and HA-PEG-ICG/PLGA NPs are 338.9 and 345 nm, respectively, as verified by TEM images, as shown in Figure 2D,2E. In addition, the average hydrodynamic diameter of these PLGA-based nanocarriers was determined through dynamic light scattering (DLS) analysis, as illustrated in Figure S5. The morphology is predominantly spherical, resembling that of the ICG/PLGA NPs. This suggests that surface modification with PEG and HA molecules does not significantly alter the morphology of the PLGA NPs. Furthermore, the fluorescence intensity and peak position of HA-PEG-ICG/PLGA NPs show minimal variation when dispersed in different solvents such as deionized (DI) water, phosphate-buffered saline (PBS), and culture medium. These nanoparticles exhibit stable behavior, making them suitable for further *in vitro* and *in vivo* studies.

**Figure 2:**
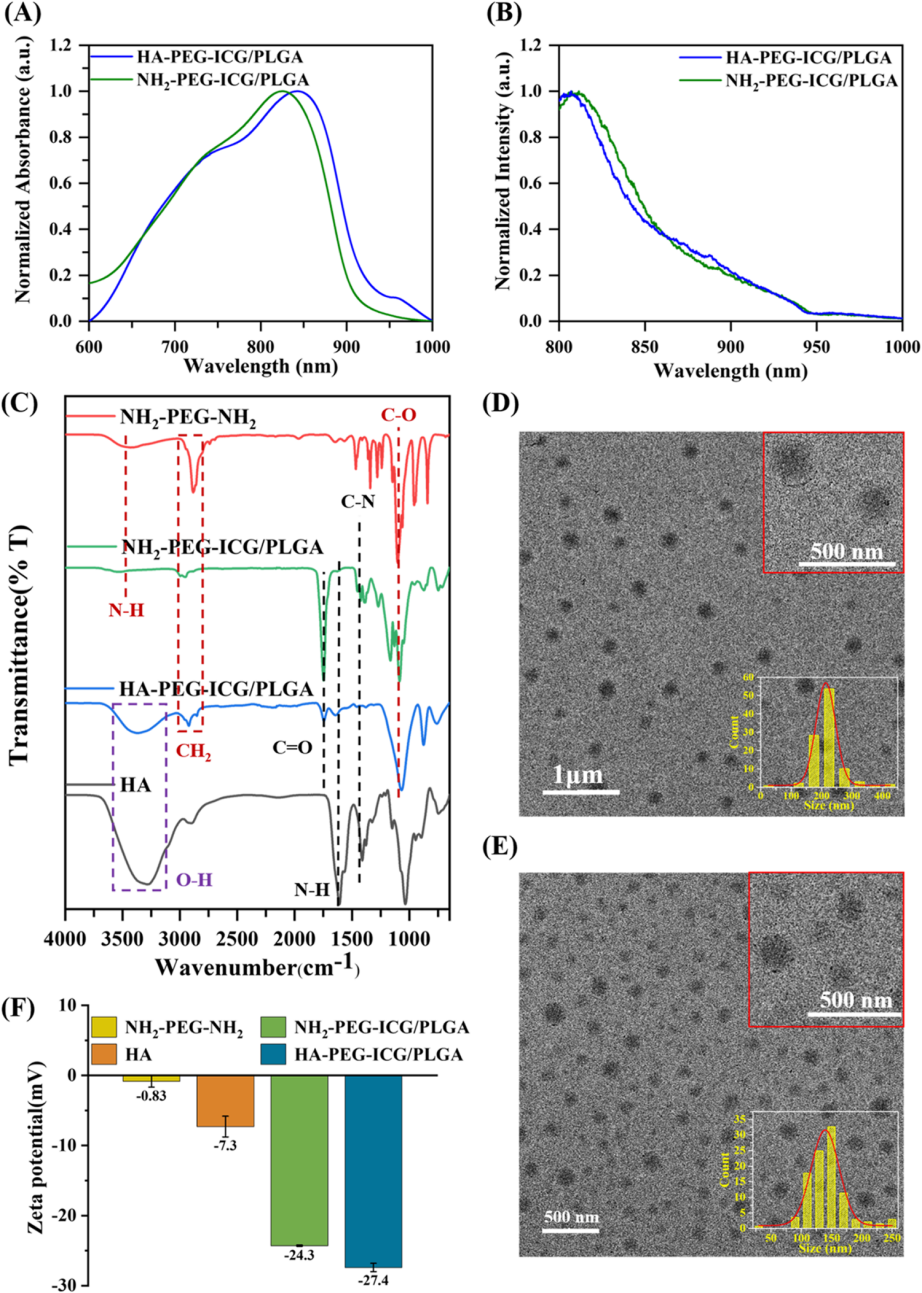
Characterization of the optical, surface functionalized group, and morphology properties of NH_2_–PEG-ICG/PLGA and HA-PEG-ICG/PLGA. (A). Normalized UV–vis spectra; (B). Normalized Photoluminescence spectra (λ_ex_ = 785 nm, CW laser, 185 mW); (C). FTIR analysis profile. TEM image of (D). NH_2_–PEG-ICG/PLGA; and (E). HA-PEG-ICG/PLGA. (F) ζ-Potential profile includes NH_2_–PEG-NH_2_, HA, NH_2_–PEG-ICG/PLGA, and HA-PEG-ICG/PLGA.

### 3.3 *In Vitro* Study: Cell Viability and Evaluation of Targeting Bioimages

To evaluate the biocompatibility of HA-PEG-ICG/PLGA NPs for future studies on lymphatic vessel targeting,HaCaT and SVEC4–10 cells were selected for cell viability assessment. The CCK-8 assay was employed to determine cell viability across various concentrations of ICG encapsulated in HA-PEG-ICG/PLGA NPs, specifically 0, 5.3, 10.5, 21, 42.1, and 84.2 μM. As shown in Figure 3A, the cell viability profiles exhibit significant biocompatible behavior at the concentrations below 42.1 μM, showing above 90% survival rate in HaCaT and SVEC4–10 cells. However, the cell viability shows dose-dependent cytotoxicity at the concentration of 84.2 μM toward HaCaT and SVEC4–10 cells, indicating an approximate survival rate of 81 ± 2.6 and 70 ± 3.9%, respectively. Thereafter, the cell-targeting capability of HA-PEG-ICG/PLGA NPs was evaluated in SVEC4–10 and HaCaT cells under a series of ICG concentrations as 10.5, 21, 42.1, 63.2, and 84.2 μM for 24 h of treatment (Figure 3B,D). Parallel experiments were conducted by using equivalent concentrations of ICG/PLGA NPs in SVEC4–10 cells (Figure 3C). The cell-targeting capability in HA-PEG-ICG/PLGA NPs was assessed through confocal microscopy, featuring with images of cell nuclei (blue color), cell membrane skeleton (green color), and ICG (red color). Notably, HA-PEG-ICG/PLGA NPs exhibit excellent specific cell-targeting performance for SVEC4–10 cells rather than HaCaT cells, indicating the fluorescence signals are localized predominantly at the cell membrane of SVEC4–10 cells (Figure 3D). In contrast, no significant fluorescence signals were detected in parallel groups (Figure 3B,3C). These results suggest that HA-functionalized NPs (HA-PEG-ICG/PLGA NPs) provide a targeting capability enhancement toward lymphatic endothelial cells. The HA molecule, which serves as an essential binding partner for LYVE-1, is produced by migrating dendritic cells (DCs) and macrophages. In this context, HA contributes to the formation of a dense glycocalyx that is anchored to the cell surface *via* CD44, a member of the hyaladherin family that shares structural similarities with LYVE-1.^34,35^ Consequently, the HA-PEG-ICG/PLGA NPs developed in this study exhibit potential as a lymphatic fluorescence probe for biomedical imaging applications.

**Figure 3:**
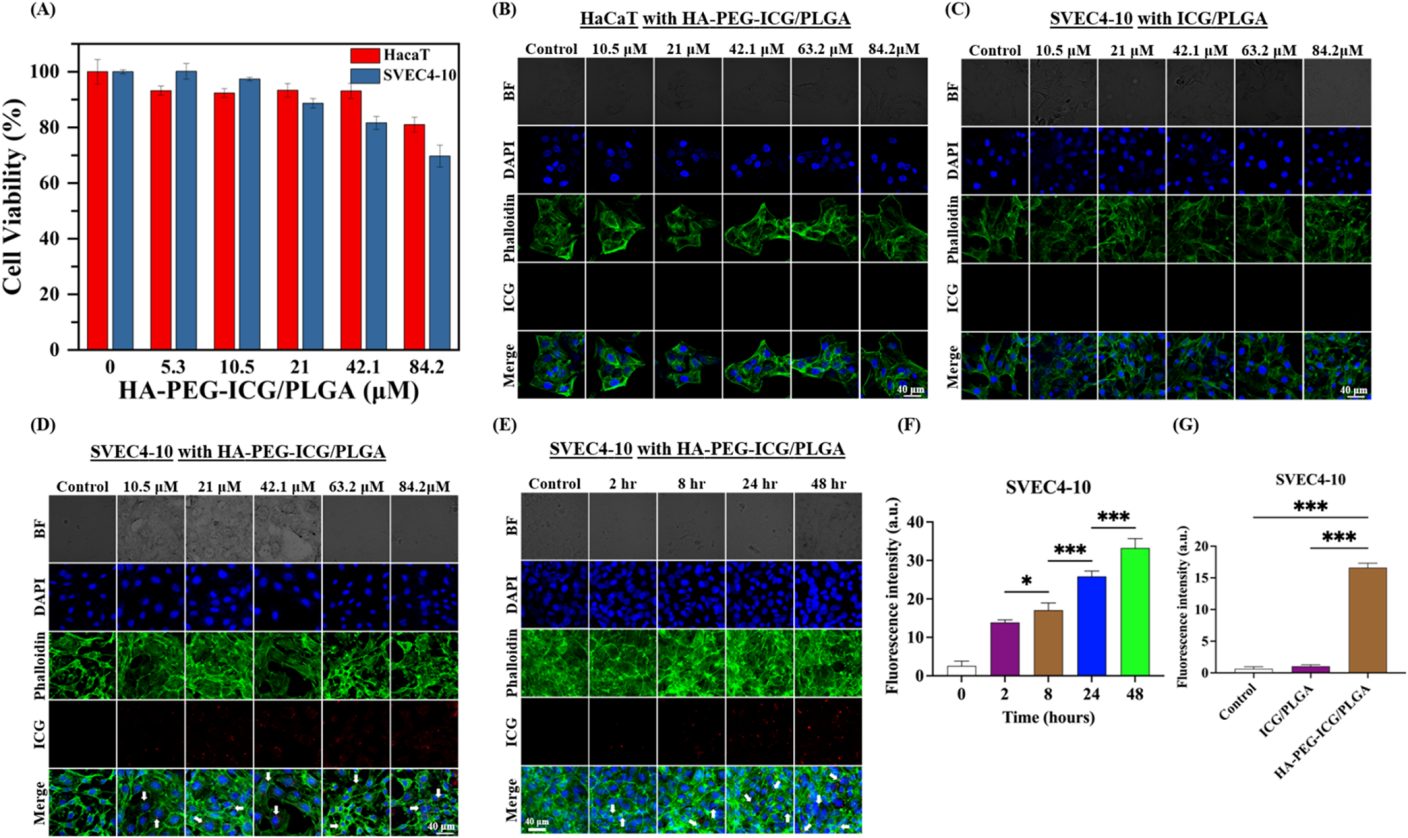
Evaluation of cytotoxicity and cellular uptake of HA-PEG-ICG/PLGA NPs and ICG/PLGA NPs in HaCaT and SVEC4–10 cells. (A) Cell viability of HaCaT and SVEC4–10 cells after incubation with HA-PEG-ICG/PLGA NPs for 24 h (*n* ≥ 4). Confocal fluorescence images of (B) HaCaT cells and (D) SVEC4–10 cells with HA-PEG-ICG/PLGA NPs for 24 h incubation (*n* ≥ 5). (C) Confocal fluorescence images of SVEC4–10 cells with ICG/PLGA NPs for 24 h (*n* ≥ 5). (E) The SVEC4–10 cells targeting efficiency represented for 2, 4, 8, 24, and 48 h with HA-PEG-ICG/PLGA NPs for 24 h incubation by confocal fluorescence images (*n* ≥ 5). (F, G) Statistical significance was determined by comparison with the results of (E) and the control group using one-way ANOVA followed by Tukey’s post hoc test. *p* values are represented as follows: *p* < 0.05 (*), *p* < 0.01 (**), and *p* < 0.001 (***). Scale bar: 40 μm. Staining agent information: Nuclei stained with DAPI (blue, excitation 405 nm, emission 430–470 nm), F-actin stained with Phalloidin (iFluor 488, green, excitation 488 nm, emission 500–540 nm), and ICG (green, excitation 640 nm, emission 650–750 nm).

### 3.4 *In Vivo* Biosafety and Lymphatic Targeting Evaluation of HA-PEG-ICG/PLGA Nanoparticles

Prior to the investigation of lymphatic vessel targeting, an *in vivo* biosafety evaluation was performed using C57BL6/6JCrlBltw mice following the intraperitoneal (IP) administration of ICG, ICG/PLGA, and HA-PEG-ICG/PLGA NPs. After a systemic circulation period of two hours, no significant alterations in body weight or histopathological abnormalities in major organs, including the heart, liver, and kidneys, were noted when compared to the PBS-treated control group (Figure 4A). These results indicate that all formulations tested demonstrate favorable *in vivo* biocompatibility, thereby affirming their potential for subsequent lymphatic imaging applications. Following this, the lymphatic targeting efficacy of HA-PEG-ICG/PLGA NPs was assessed *in vivo* by investigating the interaction between HA and the CD44/LYVE-1 receptors, which are prominently expressed on lymphatic endothelial cells.^33,21,22^ Confocal fluorescence microscopy was utilized to analyze tissue sections through bright-field imaging, ICG fluorescence, and nuclear counterstaining with Hoechst 33342 dye (Figures 4B and S6). Importantly, mice administered with HA-PEG-ICG/PLGA NPs exhibited pronounced ICG fluorescence localized to lymphatic endothelial regions, indicating specific binding of the HA component to the LYVE-1 and CD44 receptors. Conversely, minimal fluorescence signals were detected in mice treated with ICG, ICG/PLGA, or PBS, suggesting an absence of nonspecific accumulation. Collectively, these findings demonstrate that HA-PEG-ICG/PLGA NPs possess advantageous biosafety profiles and selective lymphatic targeting capabilities.

**Figure 4:**
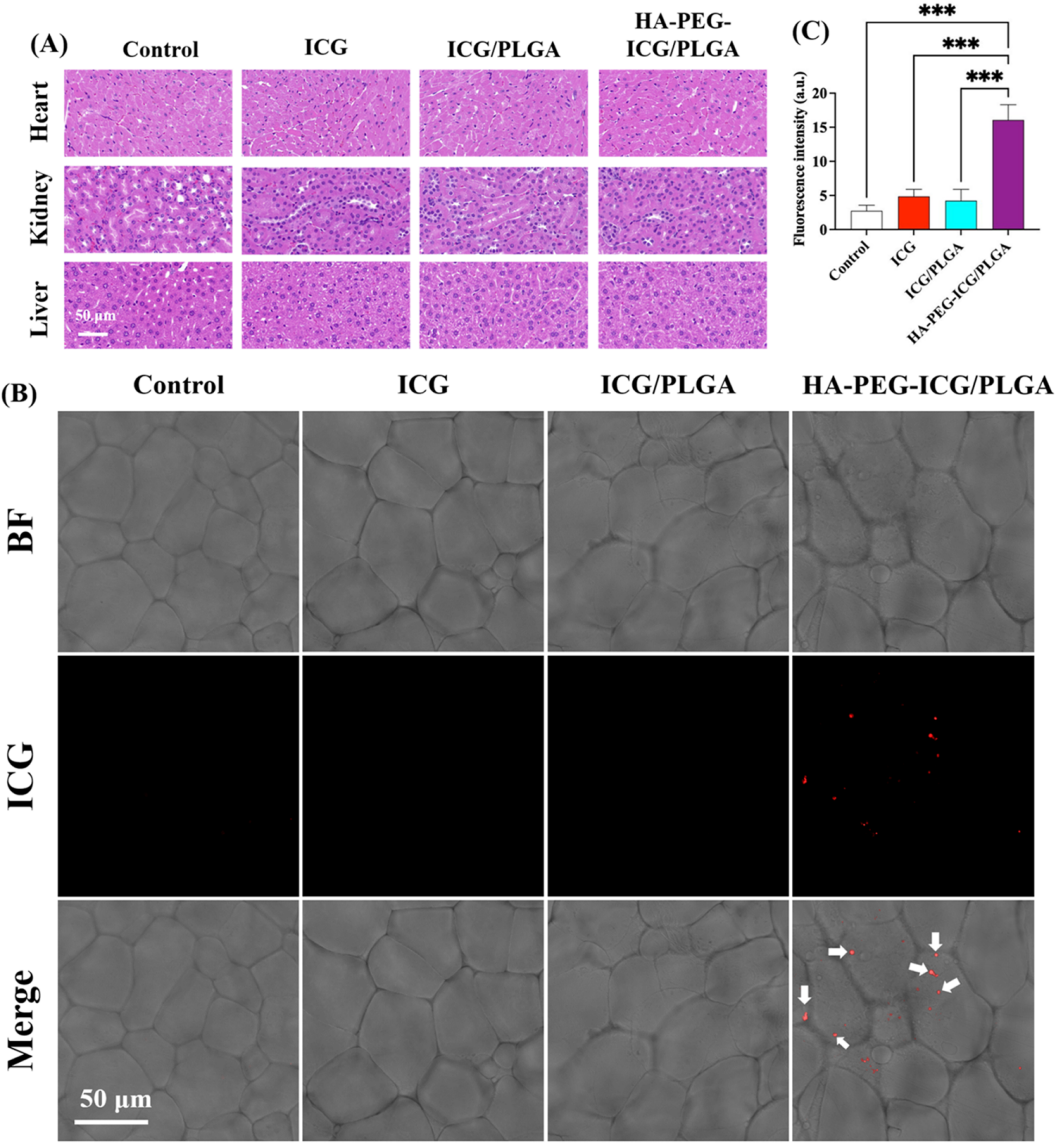
Biosafety and selective lymphatic targeting properties in C57BL6/6JCrlBltw mice model following intraperitoneal injection of ICG, ICG/PLGA, and HA-PEG-ICG/PLGA NPs (*n* = 4 for experimental condition; *n* = 3 for control). (A) Assessment of the morphology of the heart, kidney, and liver in each mouse through hematoxylin and eosin staining (scale bar, 50 μm). (B) Lymphatic targeting capability analysis of tissue sections using bright-field imaging and ICG fluorescence by confocal fluorescence microscopy (inset arrow remains parts of ICG emission, Scale bar, 50 μm). (C) Statistical significance was determined by comparison with the control group using one-way ANOVA followed by Tukey’s post hoc test. *p* values are represented as follows: *p* < 0.05 (*), *p* < 0.01 (**), and *p* < 0.001 (***).

## 4 Conclusions

In this study, we successfully introduce a microemulsion method and a surface chemistry cross-linking reaction to synthesize ICG-loaded PLGA nanoparticles functionalized with HA-PEG (HA-PEG-ICG/PLGA NPs) with a mean size of 142.6 ± 6.4 nm. We provide evidence of their characteristics through UV–vis spectroscopy, fluorescence spectroscopy, FTIR analysis, and surface ζ-potential profiles. The optical properties of absorption peaks at 744 and 843 nm. Furthermore, their emission peaks are recorded at 806 and 811 nm, respectively. *In vitro* cytotoxicity assays demonstrated high biocompatibility, with cell viability above 85% across all tested concentrations (5.3–84.2 μM ICG) in both HaCaT and SVEC4–10 cells. Quantitative confocal fluorescence imaging revealed significantly higher uptake of HA-PEG-ICG/PLGA NPs in SVEC4–10 cells compared to HaCaT cells (*p* < 0.01), attributed to HA-mediated targeting LYVE-1 receptors. *In vivo* imaging in C57BL/6JCrlBltw mice further confirmed selective lymphatic accumulation and prolonged retention, with a 2.3-fold increase in fluorescence intensity compared to non-HA-conjugated NPs at 24 h post-IP injection. These findings clearly support the selective lymphatic targeting and biosafety profile of HA-PEG-ICG/PLGA NPs. Therefore, this nanoparticle system holds strong promise as a real-time fluorescent imaging agent for lymphatic vessels and as a diagnostic platform for early stage lymphedema or related lymphatic disorders.

## Supplementary Material



## Notes

Detailed experimental processes (Figures S1–S4); UV–vis spectra of ICG with a range of ICG concentrations; linear calibration of ICG concentrations with a correlation coefficient (Figure S4); DLS analysis profile illustrates average hydrodynamic diameter of PLGA NPs, ICG/PLGA NPs, NH_2_–PEG-ICG/PLGA NPs, and HA-PEG-ICG/PLGA NPs (Figure S5); confocal fluorescence images of ICG, ICG/PLGA NPs, and HA-PEG-ICG/PLGA NPs in bright-field imaging and nuclear staining image (Figure S6) (PDF)
