# Supplementary material for: Surface-Engineered HA-PEG-ICG/PLGA Nanoprobes with Vessels Targeting for Lymphatic System Visualization
Source: ACS Appl Bio Mater. 2025 Aug 27;8(9):7783–92. doi: 10.1021/acsabm.5c00769 (PMC12442094; doi:10.1021/acsabm.5c00769)
Supplement: Supplementary file 1 [file mt5c00769_si_001.pdf]

Supporting Information

Surface-Engineered HA-PEG-ICG/PLGA Nanoprobes with vessels Targeting for Lymphatic System Visualization

Hao-Han Chiang‡[1], Yu-Teng Chang‡[3,4], Wei-Ren Huang[1], Min-Xuan Cai[1], Chin-Hsing Feng[2], Jia-Ning Syu[2], Chih-Sheng Lai[2,3], Yi-Hsin Chien[1]**

[1]Department of Materials Science and Engineering, Feng Chia University, Taichung, Taiwan

[2]Division of Plastic and Reconstructive Surgery, Department of Surgery, Taichung Veterans General Hospital, Taichung, Taiwan

[3]Department of Post-Baccalaureate Medicine, College of Medicine, National Chung Hsing University, Taichung, Taiwan

[4]Division of Nephrology, Department of Internal Medicine, Taichung Veterans General Hospital, Taichung, Taiwan

Corresponding Author

*Corresponding author e-mail: 6790pocket626@gmail.com, yhchien@fcu.edu.tw

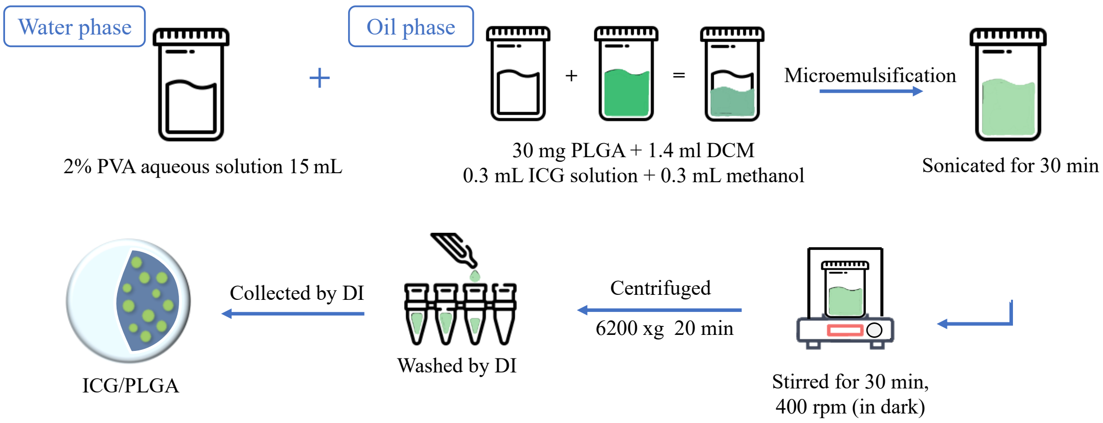

Figure S1. Synthesis of PLGA nanoparticles encapsulating ICG (ICG/PLGA NPs).

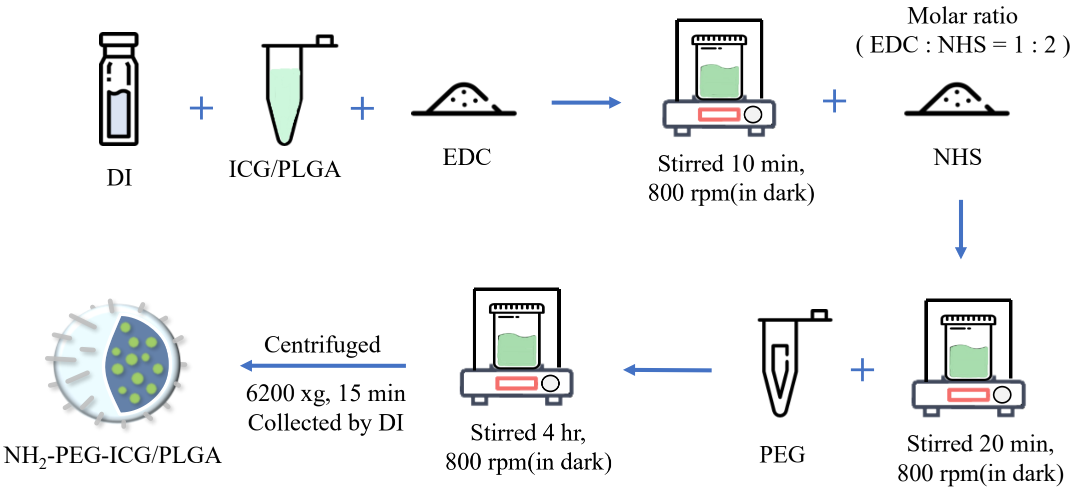

Figure S2. Synthesis of NH_2-PEG-ICG/PLGA NPs.

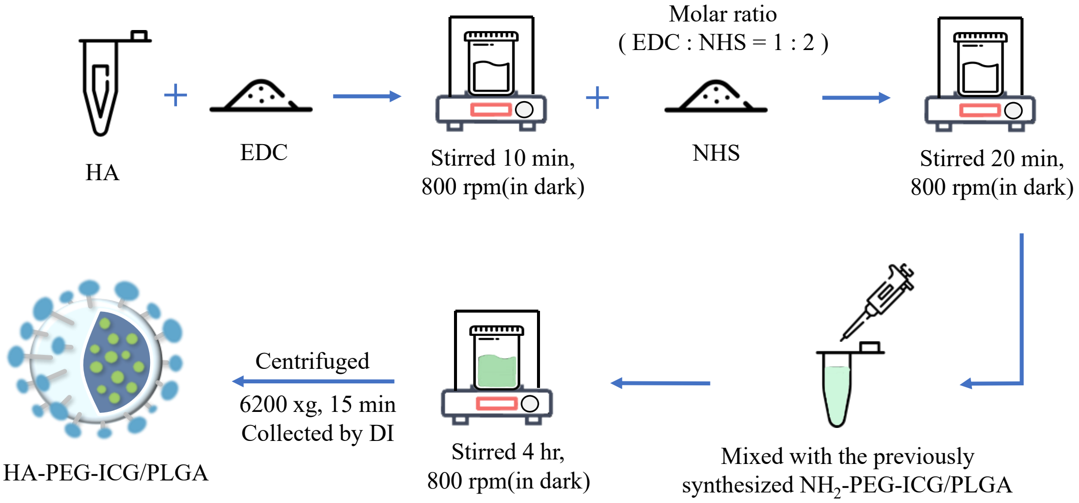

Figure S3. Synthesis of HA-PEG-ICG/PLGA NPs.

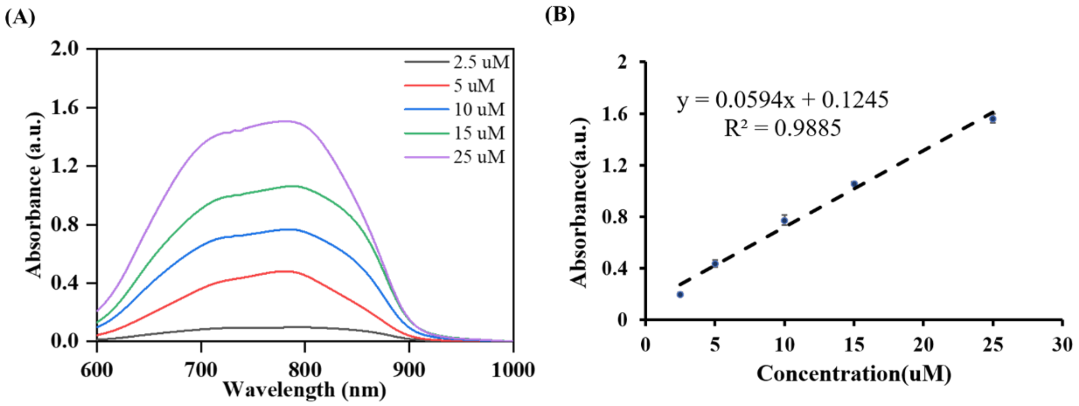

Figure S4. (A) UV-vis spectra of ICG with across a range of ICG concentrations (2.5, 5, 10, 15, and 25 µM); (B) the relationship between absorbance intensity and ICG molecule concentration range of2.5, 5, 10, 15, and 25 µM, showing a linear calibration with a correlation coefficient of R^2=0.9885.

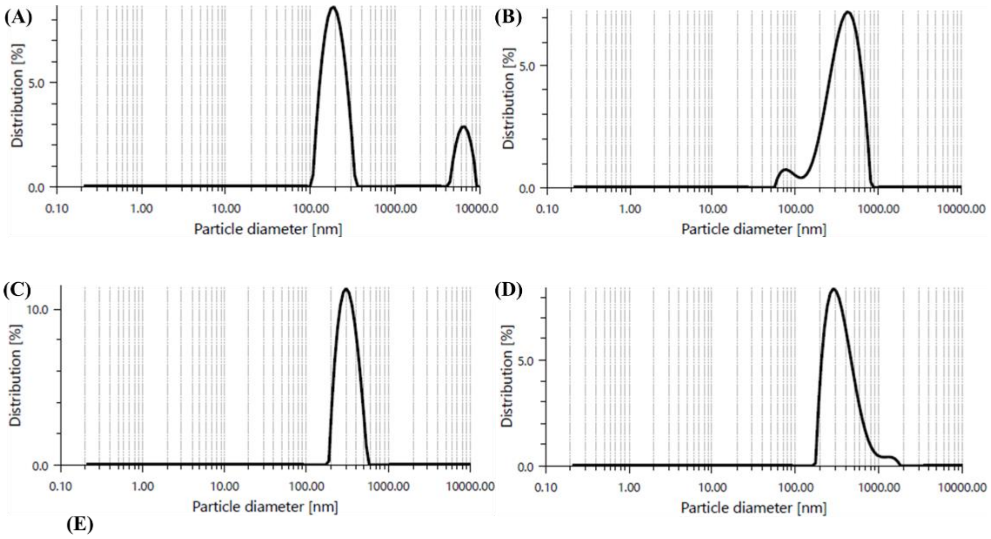

(E)

Sample	Average Hydrodynamic diameter(n=6)	Polydispersity index(PDI)
PLGA NPs	260.9 nm	10.8%
ICG/PLGA NPs	389.2 nm	24%
NH_2-PEG-ICG/PLGA NPs	338.9 nm	9.7%
HA-PEG-ICG/PLGA NPs	345 nm	20%

Figure S5. Average hydrodynamic diameter (n=6) of (A) PLGA NPs; (B) ICG/PLGA NPs; (C) NH_2-PEG-ICG/PLGA NPs; (D) HA-PEG-ICG/PLGA NPs. (E) a summarized table illustrates various hydrodynamic size and polydispersity index.

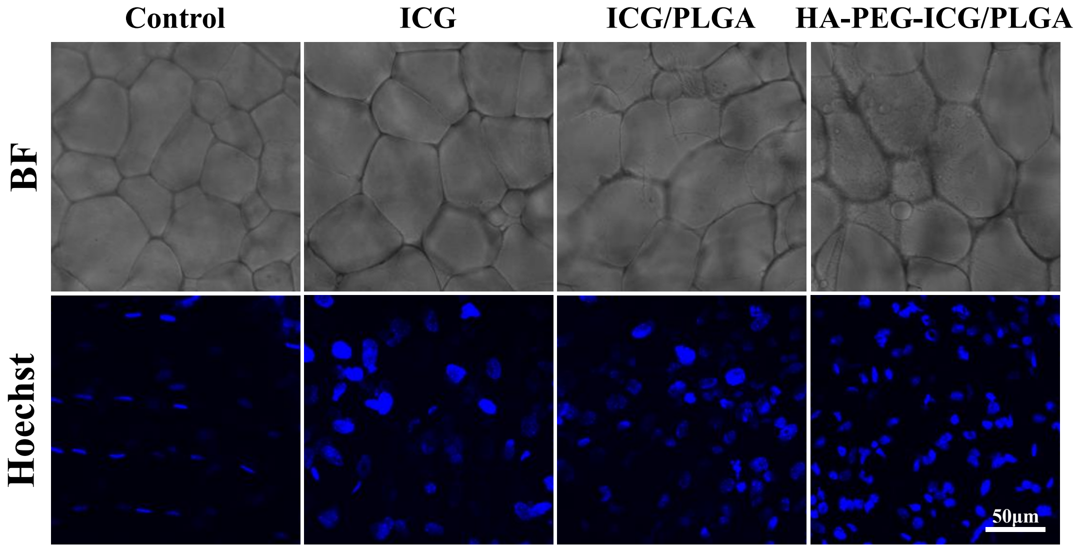

Figure S6. Confocal fluorescence microscopy was employed to analyze tissue sections using bright-field imaging and nuclear counterstaining with Hoechst 33342 dye by ICG, ICG/PLGA NPs, and HA-ICG/PLGA NPs treatment.
